# A survey of Psychiatric Disorders and Their Comorbidities in Children and Adolescents

**DOI:** 10.31661/gmj.v9i0.1714

**Published:** 2020-01-27

**Authors:** Aazam Sadat Heydari Yazdi, Mahboubeh Eslamzadeh, Mohammad Reza Mohammadi, Ali Khaleghi, Zahra Hooshyari, Fatemeh Moharreri, Seyedeh Farzaneh Ebrahimpour, Simin Ashouri, Samira Ashouri

**Affiliations:** ^1^Psychiatry and Behavioral Sciences Research Center, Mashhad University of Medical Sciences, Mashhad, Iran; ^2^Psychiatry and Psychology Research Center, Roozbeh hospital, Tehran University of Medical Sciences, Tehran, Iran

**Keywords:** Adolescents, Children, Comorbidity, Psychiatric Disorders

## Abstract

**Background:**

This community-based study aimed to investigate the psychiatric disorders and their comorbidities according to the kind of psychiatric disorders. Frequency of demographic factors and the prevalence of total psychiatric disorders in term of demographic charactheristics were obtained too.

**Materials and Methods:**

The present study focused on 1028 children and adolescent aged 6 to 18 years old across the Razavi Khorasan province by random sampling. The subjects included 496 boys and 532 girls from three age groups (6-9 years, 10-14 years and 15-18 years). Eight clinical psychologists trained to complete the Persian version of K-SADS-PL (Kiddie-SADS present and life time version). This scale measures five diagnostic appendixes of psychiatric disorders. Demographic data of participants were collected too. The data were recorded into the SPSS version 16. The relationship between psychiatric disorders and demographic factors deliberate by descriptive analysis and 95% confidence interval.

**Results:**

The total rate of psychiatric disorders among children and adolescent was estimated as 20.5%, elimination disorders with a rate of 12.9% was the most prevalent disorder in the subjects. The lowest prevalence belongs to psychotic disorder and bulimia nervosa (0.1%). Of participants with mood disorders about 71.4% have behavioral disorders too. Anxiety disorders also commonly occurred in person with mood disorders. The comparison of ORs and their 95% confidence interval revealed that there is a significant difference for total psychiatric disorder among boys and girls (OR=0.6 for girls; 95% CI: 0.44-0.82). The rate of total psychiatric disorders in rural and urban areas was 14.9% and 21.1% respectively.

**Conclusion:**

With attention to the high prevalence of psychiatric disorders among children and adolescents, it’s necessary that healthcare officials pay more attention to reinforcement of mental health care.

## Introduction

There has been a widespread need for estimating the prevalence of psychiatric disorders and recognizing the associated factors. Therefore, numerous epidemiological studies have been conducted all over the world. These studies showed different reports because of diversity in sampling methods, classifications and diagnostic tools. For example, the prevalence of mental health problems in children and adolescents estimated from 7% in rural areas of Brazil and Norway, 10% in Denmark and Britain and up to 15% in Russia and Bangladesh [1-7]. According to a recent survey, 10 to 20 percent of children and adolescents all over the world suffer from mental problems [8], Almost one third of the world’s population is children and adolescents. 90% of these people live in low and middle income countries (LMIC) where children and adolescents made up to 50% of the population. Furthermore, child and adolescent psychiatric disorders may lead to adulthood disorders and increase the suicide risk [9]. So, epidemiological surveys of mental disorders in children and adolescents play an important role to identify prevalence of disorders and developing psychiatric services in given countries. It is estimated that Iran as an LMIC had 7 milion people with psychiatric disorders. This issue shows importance of repeating epidemiological surveys during the time to recognize the trends of prevalence of psychiatric disorders for primary intervention [10]. Mohammadi and colleagues (2013) evaluated the prevalence of psychiatric problems in five provinces included Razavi Khorasan, Isfahan, Fars, Tehran and East Azerbaijan. They used self-report form of the SDQ. According to their results, conduct problems had the highest prevalence and social problems had the lowest prevalence [11]. Moharreri *et al*. (2009) conducted a study in Mashhad, Iran, 2012 children and adolescents aged from 6 to 18 years old selected from urban and rural areas of this city. The results indicated that in the self-report form of SDQ 34% of subjects had psychiatric problems, analyzing the parent form of SDQ showed that 67.7% of participants had psychiatric problems too [12]. Mohammadi *et al*. (2016) investigated the prevalence of psychiatric disorders in children and adolescents in Iran. 9636 children and adolescents aged 6- 18 years old were selected from five provinces. They found 10.55 % of children and adolescents suffer from psychiatric disorders. The highest prevalence related to oppositional defiant disorder (ODD). Among the all provinces Mashhad and Tehran had the highest prevalence of ODD [13]. The aim of our study was to identify the prevalence rate of psychiatric disorders and their comorbidity among children and adolescents in Razavi Khorasan province in order to planning and developing psychiatric services. Knowing the fact that co-factors related to the development of psychiatric disorders include gender, age, place of residence and family conditions [14], we have reported the rate of psychiatric disorders by demographic variable too.

## Materials and Methods

###  Study Design

This cross sectional, community-based study carried out in Khorasan Razavi province of Iran in 2017. NIMAD (National Institute for Medical Research Development; Grant No.940906) financially supported this study. More details about the study design and methodology could be obtained from Mohammadi *et al*. study [15].

###  Sampling

The present study selected 1028 children and adolescents aged from 6 to 18 years old across the Razavi Khorasan province by cluster and stratified random sampling. The participants included 496 boys and 532 girls in three age groups (6-9 years, 10-14 years and 15-18 years). The subjects were selected from urban and rural areas of Razavi Khorasan province. See [15] for more details about study protocol.

###  Data collection

 Eight clinical psychologists cooperated in this study.They trained to complete the persian version of K-SADS-PL (Kiddie-SADS present and life time version). Adolescents and parents of participants completed the informed consent. Then, subjects’ information form included demographic variables, socioeconomic status and education levels of parents were collected. The interview of K-SADS-PL carried out by clinical psychologists and completed by parents or participants themselves (for adolescents aged 11 or more). Each participant interviewed by two psychologists (men and women).

###  Scale

 K-SADS-PL (Kiddie-SADS-present and life time version)

The Kiddie schedule for affective disorders and schizophrenia-present and life time version (K-SADS-PL) is a instrument which applies for early and life time diagnosis of affective disorders, psychotic disorders, anxiety disorders,, eating disorders, substance abuse, disruptive behavioral disorders, tic disorders and elimination disorders [16]. Specificity and sensitivity of the Persian version of Kiddie-SADS have been assessed by Ghanizadeh *et al*. Their findings showed that this questionnaire is reliable (the reliability of this instrument in test-retest and inter-rater phases is measured as 0.81 and 0.69 respectively) [17]. Polanczyk *et al*. obtained kappa coefficients for K-SADS. The results showed kappa coefficient for affective disorders, psychosis, anxiety disorders, ADHD and disruptive behavioral disorders, elimination disorders were 0.93, 0.82, 0.93, 0.94, 0.94 respectively [18]. As mentioned, this questionnaire is valid and reliable.

###  Statistical analysis

 The data were collected across the Razavi Khorasan province. The data screening method is employed. The data were recorded into the IBM SPSS Statistics for Windows, Version 16.0 (SPSS Inc).. To measure of the relationship between psychiatric disorders and demographic variables, we used descriptive analysis, Odds ratios and 95% confidence intervals. The statistical significancy is concerned as <0.05.

###  Ethics

 The informed consent was completed by adolescent ranged in age 15 to 18 years and their parents. Parents completed the consent if participants younger than 15 years old. All information about subjects was kept confidential. The participants referred to the child and adolescent psychiatrist, who collaborated in the project, if they were diagnosed with a mental disorder for treatment out of charged. The ethics review board of NIMAD (National Institute for Medical Research Development) has approved the protocol this study (ethics code: IR.NIMAD.REC.1395.001).

## Results

**Figure 1:**
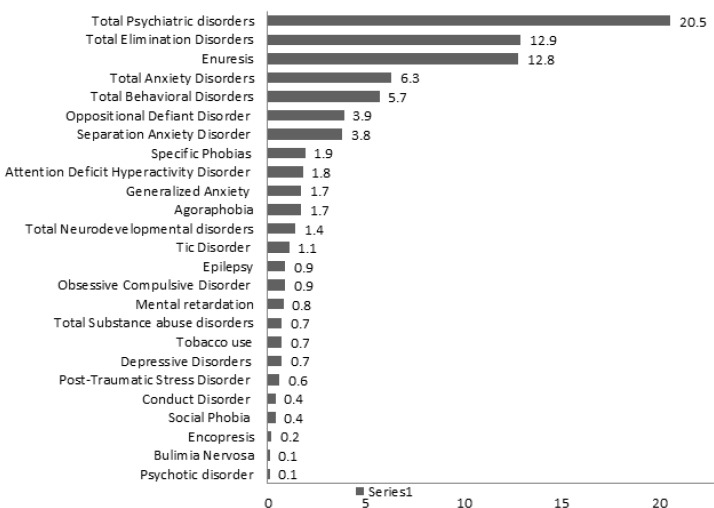
The prevalence rate of psychiatric disorders among children and adolescents in Razavi Khorasan province

 1028 children and adolescents, aged from 6 to 18 (meanSD: 11.93.79) enrolled in the present study. They were 496 (48.2%) boys and 532 (51.8%) girls from three age groups (6-9 years, 10-14 years and 15-18 years). The average of age was 11.91±3.86 years in girls and 11.89±3.72 years in boys. 90.2% of them lived in the urban and 9.8% lived in the rural areas. The most common parent education level was diploma (28.8 % of fathers and 34.9% of mothers had diploma education level) (Table-1). Table-1 shows the demographic characteristics and the total prevalence rate of psychiatric disorders in each group. Table-2 indicates comparison of odds ratio (95% confidence interval) of frequency of total psychiatric disorders in term of demographic variables. The total prevalence of psychiatric disorders among boys and girls was 24.8% and 16.5%, respectively, the comparison of 95% CI showed a significant difference for total psychiatric disorder in both genders (P=0.002). The prevalence of total psychiatric disorders among the three age groups showed 10-14 years old had the highest rate of total mental disorders (24%). The comparison of 95%CI of frequency of total psychiatric disorders in terms of age groups revealed a significant difference in 15-18 years (P=0.027). Odds ratio (95%CI) for total psychiatric disorder in term of other demographic factors doesn’t suggest significant differences statistically (Table-2). The result revealed the prevalence of total psychiatric disorders in children and adolescents was 20.5% (95% CI: 18.2-23.1). That means 211 subjects were diagnosed with at least one psychiatric disorder. It should be noted that neurodevelopmental disorders and tobacco use don’t consider in the prevalence of total psychiatric disorders. In addition Elimination disorders had the most prevalence in the participants (12.9%), the second and third prevalent disorders were anxiety disorders (6.3%) and behavioral disorders (5.7%) respectively. The most prevalent elimination disorders was enuresis (12.8%), separation anxiety disorder (3.8%) had the highest rate in total anxiety disorders. The psychotic disorder and bulimia nervosa had the lowest prevalence among all (0.1%) (Table-3). Comorbidity disorders, according to the type of psychiatric disorder in the Razavi Khorasan province is shown in Table-4. The results show that the comorbidity of mood disorders with behavioral disorders (%71.4) was more than other disorders. Comorbidity of psychotic disorders was similar with mood, anxiety and elimination disorders. Anxiety disorders were associated with behavioral disorders (29.2%) and elimination disorders (23.1%) significantly. The co-occurrence of neurodevelopmental disorders and elimination disorders were more than other disorders (28.6%).The most comorbidity of substance disorders was behavioral disorders (42.9%) (Table-4). Figure-1 shows the prevalence rate of psychiatric disorders among children and adolescents in Razavi Khorasan province.

## Discussion

This cross sectional study aimed to obtain the prevalence of psychiatric disorders an their co morbidity in children and adolescents across the Razavi Khorasan province. The results indicated that 20.5% subjects presented with at least one psychiatric disorder. The results are in agreement with presenting epidemiological data in literatures. The prevalence rate of psychiatric disorders in children and adolescents were reported as 10-20% [19], 22.5% in Switzerland and 20.7% in Germany [20], 16.3% in North Khorasan province [21] and 31.7% in Ardabil province [22]. It should be noted that the same scale (K-SADS) was used in the present study and above-mentioned studies. In Mohammadi *et al*. study, the overall prevalence of psychiatric disorders was reported 10.55% in Iran and 14.17% in Mashhad [12]. The higher rate of psychiatric disorders in the present study can be related to the time of the survey, different geography and interviewers. For example, in present study subjects were selected from urban and rural areas too, also higher prevalence of psychiatric disorders in the present study may be due to the growing prevalence of psychiatric disorders over the time. Child and adolescent mental health estimated that 10-20% of child and adolescent suffered from psychiatric disorders which was very close to our results [23,24]. The epidemiological survey of psychiatric disorders was conducted by Moharreri *et al*. in Mashhad city (2009) showed that 34% of subjects are affected by psychiatric problems in the self-report form of SDQ [11], the difference between two studies can be related to different scales used for screening and diagnosis of psychiatric disorders, for example, SDQ is a screening tool for investigating of behavioral and emotional problems, it could estimate a higher prevalence relative to a diagnostic scale such as K-SADS. The most prevalent disorder was elimination disorders (12.9%) in our study. Enuresis had the highest rate between elimination disorders (12.8%). Similarly, Torkashvandand *et al*. have reported 10.6% enuresis among children in Rafsanjan [25]. The frequency of anxiety and behavioral disorders was 6.3% and 5.7%, respectively. Separation anxiety disorder (3.8%) and oppositional defiant disorder (3.9%) had the highest rate in total anxiety and behavioral disorders. Zarafshan *et al*. performed a systematic review in Iran to assess the prevalence of anxiety disorders in children and adolescents, the result of their study indicated that the prevalence of separation anxiety disorder was 0.7%-15.7% that supports the results of the present study. They also showed the rate of generalized anxiety disorder (0.54% to 12.8%) and Obsessive compulsive disorder (1% to 11.9%) which approximately confirms our result [26]. Also Solhdoost and *et al*. have reported 12.1% sever anxiety [27]. In a study conducted by Mohammadi *et al*. Oppositional defiant disorder (ODD) was the most prevalent psychiatric disorders in five provinces of Iran (4.45%) [13]. Although the ODD didn’t have the highest rate in our study, but with a rate of 3.9% was one of the most prevalent disorders. The prevalence rate of psychotic disorders and bulimia nervosa in our study was 0.1%, which consistent with the result of previous study conducted in Iran [13]. The present study also revealed that the total frequency of psychiatric disorders had decreasing trend from boys to girls. The comparison of OR for prevalence of psychiatric disorders in term of sex showed a significant difference between two genders (P=0.002) which is consistent with the results of other literature [6,13,20]. Our study showed the place of residence also affects on the prevalence of psychiatric disorders. The prevalence of total psychiatric disorders in urban was more than rural environments, this finding suggests that social stressor in urban setting affect the development of psychiatric disorders. The results indicated that the comorbidity of mood disorders with behavioral disorders (%71.4) and anxiety disorders (28.6%) were more than the other disorders. This finding supports the results of the previous studies. For examples, Tonna *et al*. (2015) showed that approximately 50% of bipolar patients have at least one other psychiatric disorders. Anxiety disorders were one of the most comorbidity in their study [28]. Many case reports suggest that mood disorders may co occure with anxiety disorders and behavioral disorders [29]. Comorbidity of depression disorders and behavioral disorders reported by some researchers too [30].

## Conclusion

 The findings of the present study revealed that the rate of total psychiatric disorders in children and adolescents in Razavi Khorasan province was estimated as 20.5%. Elimination disorders and anxiety disorders were the most prevalent disorders (12.9% and 6.3% respectively). The psychotic disorder and bulimia nervosa had the lowest prevalence among all (0.1%). The results indicated that the comorbidity of mood disorders with behavioral disorders (%71.4) were more than the other disorders. With attention to the growing prevalence of psychiatric disorders and burden of these disorders, it’s necessary that healthcare officials pay attention to improvement of mental health care system.

## Limitations

 The main limitation of the study was that this survey was carried out in urban and rural areas of Mashhad city as the representative of other cities of the province.

## Acknowledgement

 The authors would like toexpress their sincere gratitude to the National Research Institute for Medical Research, NIMAD (Grant Number: 940906), theMashhad University of Medical Sciences, the Psychiatry and Psychology Research Center,Tehran University of Medical Sciences, and all the participants of this study.

## Conflict of interest

 No conflict of interest.

**Table 1:** Frequency of Demographic Variables in Children and Adolescents (6-18) of Razavi Khorasan Province and Prevalence of Psychiatric Disorders in Terms of These Variables

		**total**		**With disorder**		**CI (95%)**
		**N**	**P**	**n**	**p**	
**Sex**	Boy	496	48.2	123	24.8	0.21-0.29
	Girl	532	51.8	88	16.5	0.14-0.20
**Age (Year)**	6-9	350	34	76	21.7	0.18-0.26
	10-14	342	33.3	82	24	0.20-0.29
	15-18	336	32.7	53	15.8	0.13-0.20
**Place of residence**	Urban	927	90.2	196	21.1	0.19-0.24
	Rural	101	9.8	15	14.9	10-23
**Fathers educational level**	Illiterate	52	5.1	6	11.5	5-23
	Primary school	207	20.2	52	25.1	19.7-31.4
	Guidance & high school	235	23	50	21.3	16.5-26.9
	Diploma	295	28.8	58	19.7	15.5-24.6
	bachelor	181	17.7	34	18.8	13.8-25.1
	M.Sc. or higher	53	5.2	11	20.8	12-33.4
	Missing	5	-	0		
**Mothers educational level**	Illiterate	46	4.5	3	6.5	2.24-17.5
	Primary school	230	22.5	54	23.5	18.5-29.4
	Guidance & high school	189	18.5	41	21.7	16.4-28.1
	Diploma	356	34.9	73	20.5	16.6-25
	bachelor	169	16.6	33	19.5	14.3-26.2
	M.Sc. or higher	31	3	5	16.1	7-32.6
	Missing	7	-	2		
**Fathers job**	Public sector	250	24.3	53	21.2	16.6-26.7
	Private sector	728	70.8	150	20.6	17.8-23.7
	unemployed	41	4	5	12.2	5.3-25.6
	Missing	9		3		
**Mothers job**	Public sector	79	7. 7	11	13.9	8-23.2
	Private sector	31	3	9	29	16-46.6
	unemployed (Housewife)	913	88.8	189	20.7	18.2-23.4
	Missing	5		2		
	**Total**	1028	100	211	20.5	18.2-23.1

**Table 2:** Odds Ratios (95% CI) for Total Psychiatric Disorder in Term of Demographic Variables

	**Variables and their categories**		**OR (crude)**	**CI (95%)**	**P-value**	**OR (adjusted)**	**CI (95%)**	**P-value**
**Demographic variables **	**Sex**	Male	1.00 Baseline					
		Female	0.601	0.442-0.816	0.001	0.603	0.440-0.827	0.002
	**Age group**	6-9 years	1.00 Baseline					
		10-14 years	1.137	0.797-1.622	0.479	1.093	0.756-1.582	0.636
		15-18 years	0.675	0.458-0.995	0.047	0.627	0.415-0.949	0.027
	**Locus of life**	Urban	1.00 Baseline					
		Rural	0.651	0.368-1.151	0.140	0.565	0.306-1.042	0.068
	**Father education**	Illiterate	1.00 Baseline					
		Primary school	2.572	1.039-6.370	0.041	1.648	0.556-4.880	0.367
		High school	2.072	0.837-5.129	0.115	1.121	0.364-3.449	0.842
		Diploma	1.876	0.764-4.605	0.170	0.993	0.313-3.158	0.991
		Bachelor	1.773	0.700-4.489	0.227	0.883	0.252-3.086	0.845
			M.Sc. or higher	2.008	0.683-5.907	0.205	0.879	0.207-3.725	0.861
	**Mother education**	Illiterate	1.00 Baseline					
		Primary school	4.398	1.312-14.740	0.016	3.014	0.810-11.210	0.100
		High school	3.971	1.172-13.455	0.027	3.095	0.794-12.060	0.103
		Diploma	3.697	1.115-12.255	0.032	2.777	0.708-10.888	0.143
		Bachelor	3.478	1.016-11.906	0.047	2.903	0.685-12.298	0.148
		M.Sc. or higher	2.756	0.608-12.501	0.189	1.641	.269-10.032	0.592
	**Father job**	Public sector	1.00 Baseline					
		Private sector	0.965	0.678-1.372	0.841	0.737	0.471-1.153	0.181
		unemployed	0.516	0.193-1.380	0.188	0.595	0.202-1.751	0.346
	**Mother job**	Public sector	1.00 Baseline					
		Private sector	2.529	.927-6.899	0.070	2.105	0.731-6.059	0.168
		Unemployed (Housewife)	1.614	0.837-3.112	0.153	1.457	0.688-3.086	0.326

**Table 3:** Prevalence of Psychiatric Disorders in the Razavi Khorasan province children and adolescents (6-18)

**Psychiatric Disorders**		**Number **	**Percent**	**CI (95%)**
**Depressive Disorders**	7	0.7	0.3-1.4
**Psychotic disorder**	1	0.1	0.02-0.5
**Anxiety disorders**	Separation Anxiety Disorder	39	3.8	2.78-5.14
	Social Phobia	4	0.4	0.15-1
	Specific Phobias	20	1.9	1.3-3
	Agoraphobia	17	1.7	1.03-2.63
	Generalized Anxiety disorder	17	1.7	1.03-2.63
	Obsessive Compulsive Disorder	9	.9	0.5-1.7
	Post-Traumatic Stress Disorder	6	.6	0.3-1.3
	Total Anxiety Disorders	65	6.3	5-8
**Behavioral Disorders**	Attention Deficit Hyperactivity Disorder	18	1.8	1.1-2.8
	Oppositional Defiant Disorder	40	3.9	2.9-5.3
	Conduct Disorder	4	.4	0.15-1
	Tic Disorder	11	1.1	0.6-1.9
	Total Behavioral Disorders	59	5.7	4.5-7.3
**Neurodevelopmental disorders**	Mental retardation	8	0.8	0.4-1.5
	Epilepsy	9	0.9	0.5-1.7
	Total Neurodevelopmental disorders	14	1.4	0.8-2.3
**Substance abuse disorders**	Tobacco use	7	0.7	0.3-1.4
	Total Substance abuse disorders	7	0.7	
**Elimination Disorders**	Enuresis	132	12.8	10.9-15.02
	Encopresis	2	0.2	0.05-0.7
	Total Elimination Disorders	133	12.9	11-15.1
	**Bulimia Nervosa**	1	0.1	0.02-0.6
	**Total Psychiatric disorders**	211	20.5	18.2-23.1

**Table 4:** Comorbid psychiatric disorders according to the type of psychiatric disorder in the Razavi Khorasan province

**Comorbid disorder** ** Main disorder**	**Eating Disorder**	**Elimination Disorders** **F(P)**	**Substance abuse disorders** **F(P)**	**Neuro developmental disorders** **F(P)**	**Behavioral Disorders** **F(P)**	**Anxiety Disorders** **F(P)**	**Psychotic disorders**	**Mood Disorders** **F(P)**
**Mood Disorders**	1(14.3)	2(28.6)	1(14.3)	0	5(71.4)	2(28.6)	1(14.3)	
**Psychotic** **disorders**	0	1	0	0	1	1		1
**Anxiety Disorders**	0	15(23.1)	2(3.1)	1(1.5)	19(29.2)		1(1.5)	2(3.1)
**Behavioral Disorders**	1(1.7)	19(32.2)	3(5.1)	1(1.7)		19(32.2)	1(1.7)	5(8.5)
**Neurodevelopmental disorders**	0	4(28.6)	0		1(7.1)	1(7.1)	0	0
**Substance abuse disorders**	0	1(14.3)		0	3(42.9)	2(28.6)	0	1(14.3)
**Elimination Disorders**	0		1(0.8)	4(3)	19(14.3)	15(11.3)	1(0.8)	2(1.5)
**Eating Disorder**		0	0	0	1	0	0	1
